# Surgery

**Published:** 1902-12

**Authors:** James Swain


### SURGERY.

**The surgical treatment of chronic Bright's disease** is one of the latest and most startling announcements, and if the claims of cure reported by certain surgeons are substantiated the treatment would be welcomed as a hopeful feature in the conduct of an intractable and dangerous malady.

Dr. Schmitt[2] discusses the question from a historical and clinical point of view, and his paper is an opportune *resumé* of the work that has been accomplished.

Reginald Harrison,[3] in 1896, reported some cases in which the kidney was exposed under the impression that a calculus or pus was present. The exploration, however, failed to discover any obvious cause for the symptoms of colicky pain, hæmaturia and albuminuria; but the operation was followed by a complete disappearance of all the symptoms. He attributed the symptoms mentioned to an increased intra-renal tension consequent upon congestion and inflammation within the kidney.

Doubtless this explanation is correct in some instances, as

*Med. Rec.*, 1902, lxii. 401. [3] *Lancet*, 1896, i. 18.

in a case of suppression of urine associated with varicella, upon whom I unsuccessfully performed nephrotomy some months ago In other cases, however, I believe the symptoms are due to an undiscovered mobile kidney which has subsequently become fixed as a result of the tearing of the tissues accompanying the exposure of the kidney. Of such cases I have had several; and this appears to me to be the most satisfactory explanation of the disappearance of the symptoms after operation.

As a result of his observations, Reginald Harrison [1] advocated either reni-puncture or capsular nephrotomy (division of the proper renal capsule) under the following conditions: (1) Suppression of urine with alarming symptoms, as in scarlatinal nephritis; (2) Progressive signs of kidney degeneration, as shown by the persistence or increase of albumin; (3) Where a marked disturbance of the heart and circulatory apparatus arises in the course of inflammatory renal disorders. Although in these cases both kidneys are probably involved, operation upon one kidney is sufficient on account of the sympathetic connection between the two organs.

Israel,[2] who has had a similar experience in the relief of symptoms following the exploration of the kidney in cases of colic and hæmaturia where no calculus was found, explains the disappearance of the renal symptoms, though only one kidney was operated upon, by assuming the existence of a unilateral nephritis. He distinctly disavows any intention to treat chronic Bright's disease or nephritis surgically, and only attacks these diseases when the hæmaturia and colic are traceable to one side only.

Edebohls [3] noticed that patients afflicted with movable kidney and chronic nephritis at the same time were completely relieved of both conditions by operative fixation of the kidney. He concluded that the displacement of the kidney was directly responsible for the existence of Bright's disease, and, therefore, placed chronic nephritis on the list of indications for nephropexy. More recently, however, he has advanced the theory that the curative effect of nephropexy upon chronic Bright's disease is brought about by an adhesive inflammation, which in establishing collateral circulation supplies the diseased kidney with new blood-vessels. The presence or absence of renal mobility is irrelevant, and is of importance only so far as to change the method of operation: in the former nephropexy, in the latter bilateral decortication, is resorted to. Edebohls also believes that unilateral nephritis is of frequent occurrence.

Pousson[4] holds somewhat similar views and, like Reginald Harrison, attributes the disappearance of hæmaturia and nephralgia to the decrease of intra-renal tension after cortical incision.

Schmitt thinks it an occasion for regret that Edebohls draws

[1] *Brit. M. J.*, 1901, ii. 1126. [2] *Deutsche med. Wchnschr.*, 1902, No. 2.
[3] *Med. Rec.*, 1901, lix. 690. [4] *Association Française d'Urologie*, Oct., 1899.

his deductions from observations on movable kidneys. Displacement of the kidney, in common with many other local processes, may be associated with the presence of albuminuria and casts, but yet have not the significance of chronic Bright's disease.

Even if chronic Bright's disease actually exists, we should not hastily conclude that the disease has been cured because albuminuria disappears after decortication of the capsule of the kidney. The symptoms of Bright's disease are sometimes intermittent; and I have performed appendicectomy upon a patient who had suffered from chronic Bright's disease for many years, in whom the albuminuria temporarily disappeared after the operation on the appendix. It would, however, be a *reductio ad absurdum* to recommend the removal of the appendix for the relief of chronic Bright's disease.

Schmitt thinks that operation in chronic Bright's disease represents only a symptomatic and not a curative measure, and from a study of the published records formulates the following conclusions: (1) In acute infectious diseases, anuria with uræmic symptoms threatening the life of the patient can be successfully combated by capsular incision or renal cleavage, which relieves congestive swelling and excess of intra-renal pressure. Operation on one side is sufficient to bring about an abundant urinary secretion, followed by a subsidence of the alarming general features. (2) Anuria with uræmic symptoms occurring in the course of chronic Bright's disease, has been temporarily relieved in some cases by surgical procedures, but in no case has a permanent cure been thereby effected. (3) When the kidney has been operated upon directly for the cure of chronic Bright's disease, the outcome has been a failure. (4) In exceptional cases, colicky pains and hæmaturia are caused by chronic Bright's disease. Capsular incision or cleavage of that kidney to which the disturbances were traceable, has been attended by excellent results. There can be no doubt that when medical expedients have failed, surgical interference has succeeded in checking the hemorrhages and alleviating the pains, but it does not inhibit the process of chronic Bright's disease. (5) Nephropexy may cure the ailments incident to movable kidney; it may remove albuminuria, if this be the result of local irritation, consequent upon the displacement; if, however, the movable kidney is affected by chronic Bright's disease, this affliction will remain uninfluenced by operation.

From these observations it is clear that a careful selection must be made of cases suitable for surgical interference. The subject cannot perhaps be said to be definitely settled, but it is well to remember that albuminuria and Bright's disease are not synonymous terms.

**Pneumococcic arthritis** is a disease of some rarity, but Herrick [1] has collected a good many cases and has brought out

[1] *Am. J. M. Sc.*, 1902, cxxiv. 12.

the chief points in its diagnosis and treatment. Herrick's observations may be regarded as an amplification of an able paper by Dr. E. J. Cave,[1] who was one of the first writers on this subject.

No age appears to be exempt, but it occurs more often in adult males. It usually arises during convalescence from pneumonia, and its clinical manifestations may be described as those common to all acute inflammations of joints. The pain varies in severity from that which is slight and has come on gradually to that which is of extreme severity and abrupt onset. The joint is tender and swollen, and fluctuation can be detected when the accumulation of fluid is great. There is also in many cases peri-articular involvement, as shown by the redness, superficial tenderness, and widespread œdema. Under these circumstances the appearance of the joint is not unlike that seen in many cases of gonorrhœal arthritis.

As regards the diagnosis in cases with much peri-articular swelling, it is to be remembered that the inflammation and the resulting abscess may be near or about the joint, and yet not involve the joint proper.

The occurrence of an arthritis during the course o a pneumonia, and especially during convalescence from the same, would naturally lead to the suspicion of a pneumococcic inflammation, especially if the patient were an alcoholic, or if his affected joint were the seat of a previous trauma or injury from rheumatism or gout; but inasmuch as an arthritis complicating pneumonia may be due to the presence of other organisms, it becomes necessary to make an exploratory puncture of the joint, and, by culture, to determine as to the presence of the pneumococcus and its degree of virulence.

The constitutional symptoms show great variation. High fever, rapid pulse, dry tongue, delirium—in a word, the clinical picture of a severe septicæmia may be present.

The pneumococcus reaches the joint through the blood current in which the organisms have been demonstrated. The factor that determines the involvement of a particular joint is not always clear, yet experimentally it has been shown that a joint previously damaged is far more likely to be the seat of the local action of the pneumococcus when it has been injected into the blood than one that has not been injured.

The changes that occur in the joint vary largely with the duration of the inflammation, and also undoubtedly with the virulence of the micro-organisms, as well as the resisting power of the body in general, and in particular the joint structures. In the acute cases often little more is seen than a synovitis with redness and swelling of the synovia, and the ordinary histological changes accompanying such a condition. The exudate varies from a serous or sero-fibrinous fluid to the more commonly found thick, yellowish pus, which contains the

[1] *Lancet*, 1901, . .

pneumococcus. More than one joint may be affected, and one joint may contain a purulent, and another a serous, exudate. In other cases, and particularly those of a more subacute or chronic character, the changes are more extensive and destructive. The cartilage may be crowded, the bone invaded, the ligaments destroyed, the tendon sheaths and muscles in the vicinity of the joint involved, so that the inflammation is a peri-arthritis as well as an arthritis. In the cases of longer-standing attempts at regeneration are made, and healing may occur with more or less deformity from bony outgrowths and from the adhesions of scar tissue.

The larger joints are most often involved, and the knee is the joint most frequently affected.

The prognosis is grave—mortality, 65 per cent.—largely because of the accompanying bacteriæmia and involvement of other more vital parts of the body (meninges, pleura, pericardium, etc.). Yet spontaneous recovery occasionally follows, even where there is a purulent exudate.

The well-known fact that pneumococcic infections of other structures are often relatively benign is suggestive that the treatment in these cases might be less radical than in other cases of pyarthritis. Where, however, the fluid is purulent, Herrick thinks it wise to incise at once and drain the joint; but he thinks it questionable whether numerous drainage tubes or gauze drains, with free irrigation—in other words, whether too much manipulation of the joint—is advisable.

His conclusions are, that while the prognosis, for the reasons above stated, must always be uncertain and generally grave; and while, with pus in the joint, an early, free incision is to be advocated, it is well to remember that with serous exudate, and in a few cases even with purulent exudate, recovery has ensued either spontaneously or by the simpler measures of rest, compression, or aspiration; and he believes that in the arthrotomy, which should be done early when it is indicated, there should be as little damage as possible to the joint structures by unnecessary manipulation.

In this connection we may call to mind the well-known fact that some cases of empyœma due to pneumococcus infection are cured by simple aspiration.

James Swain.

